# The mGlu2/3 Receptor Agonists LY354740 and LY379268 Differentially Regulate Restraint-Stress-Induced Expression of c-Fos in Rat Cerebral Cortex

**DOI:** 10.1155/2013/736439

**Published:** 2013-11-19

**Authors:** M. M. Menezes, M. A. Santini, M. J. Benvenga, G. J. Marek, K. M. Merchant, J. D. Mikkelsen, K. A. Svensson

**Affiliations:** ^1^Neuroscience Discovery, Eli Lilly & Company, Indianapolis, IN 46285, USA; ^2^Neurobiology Research Unit, Copenhagen University Hospital Rigshospitalet, 2100 Copenhagen, Denmark; ^3^Abbott Laboratories, Global Pharmaceutical Research and Development, Neuroscience Clinical Development, Abbott Park, IL 60064-6075, USA

## Abstract

Metabotropic glutamate 2/3 (mGlu2/3) receptors have emerged as potential therapeutic targets due to the ability of mGlu2/3 receptor agonists to modulate excitatory transmission at specific synapses. LY354740 and LY379268 are selective and potent mGlu2/3 receptor agonists that show both anxiolytic- and antipsychotic-like effects in animal models. We compared the efficacy of LY354740 and LY379268 in attenuating restraint-stress-induced expression of the immediate early gene c-Fos in the rat prelimbic (PrL) and infralimbic (IL) cortex. LY354740 (10 and 30 mg/kg, i.p.) showed statistically significant and dose-related attenuation of stress-induced increase in c-Fos expression, in the rat cortex. By contrast, LY379268 had no effect on restraint-stress-induced c-Fos upregulation (0.3–10 mg/kg, i.p.). Because both compounds inhibit serotonin 2A receptor (5-HT_2A_R)-induced c-Fos expression, we hypothesize that LY354740 and LY379268 have different *in vivo* properties and that 5-HT_2A_R activation and restraint stress induce c-Fos through distinct mechanisms.

## 1. Introduction

Preclinical and clinical studies indicate that modulation of glutamatergic activity in the brain may have therapeutic value for the treatment of schizophrenia and anxiety-related disorders [1, 2]. Glutamate acts through ligand-gated ion channels and G-protein-coupled metabotropic glutamate (mGlu) receptors. The mGlu receptors can be subdivided into three groups (Group I: mGlu1, 5; Group II: mGlu2, 3; Group III: mGlu4, 6, 7, 8) based on the sequence homology, signal transduction pathways, and pharmacology [3, 4]. Activation of presynaptic mGlu2 receptors with mGlu2/3 agonists negatively modulates the release of glutamate providing a feedback that prevents excessive glutamate release [5, 6]. Presynaptic mGlu2/3 receptors also regulate the release of other neurotransmitters [7], and postsynaptic mGlu2/3 receptors can regulate neuronal excitability via the modulation of ion channel functions [5].

The actions of multiple mGlu2/3 agonists and mGlu2 positive allosteric modulators (PAMs) have been explored in animal models predictive of antipsychotic and anxiolytic activity. Of these, the two orthosteric mGlu2/3 agonists, LY354740 and the structurally related compound LY379268, have been widely studied. LY354740 and LY379268 block PCP- and amphetamine-induced hyperlocomotion [8], two commonly used models of the positive symptoms of schizophrenia. Both compounds also show efficacy in alleviating cognitive deficits induced by PCP. For example, LY354740 improved the detrimental effects of PCP on the performance in a T-maze task [9], while LY379268 attenuated a PCP-induced cognitive deficit in the 5-choice serial reaction task [10].

In anxiety models, however, some studies have shown that the properties of LY354740 and LY379268 differ. LY354740 showed activity in a wide variety of anxiety models such as fear-induced potentiated startle [11], elevated plus maze [11, 12], and stress-induced hyperthermia and improved the symptoms of patients with generalized anxiety disorder with similar efficacy as a benzodiazepine comparator [13]. However, although only a few reports have evaluated the anxiolytic properties of LY379268, the compound does not have identical effects. While LY379268 reduced stress-induced hyperthermia [14] and inhibited immobilization-induced hyperprolactinemia [15], it had no effect on the elevated plus maze [14] and might even have anxiogenic-like properties in some models, as it increases startle reflex magnitude [16]. A recent publication supported these observations where LY379268 at a dose of 3 mg/kg was found to show anxiogenic-like behavior in the light dark box and open field test [17].

The aim of the present study was to evaluate further the effect of LY354740 and LY379268 in animal models relevant to both anxiolytic and antipsychotic activity. The first part of the study evaluated the efficacy of LY354740 and LY379268 by assessing the modulation of restraint-stress-induced neuronal activation as measured by c-Fos protein. Extensive characterization of restraint-stress-induced c-Fos expression in rat forebrain has previously been reported [18–21] and the suppression of stress-induced c-Fos expression in the rat brain has been associated with the anxiolytic effects of benzodiazepines in the fear-conditioning model of anxiety [22].

**Figure 1:**
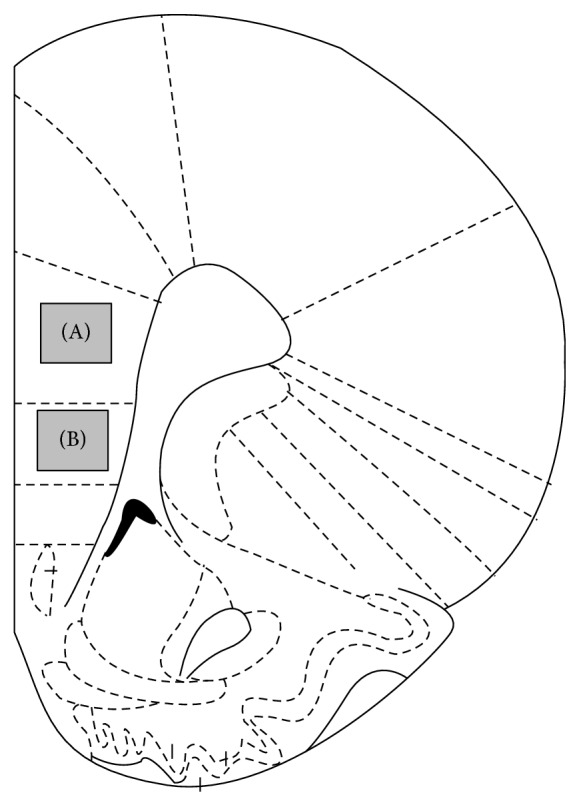
Schematic diagrams of coronal brain sections at Bregma + 2.7 mm according to the atlas [27]. Fos-positive cells were counted within the gray frame for the prelimbic cortex (A) or the infralimbic cortex (B).

One mechanism through which this class of compounds is considered to act is via negative modulation of serotonin 2A receptor (5-HT_2A_R)-dependent signaling. 5-HT_2A_R and mGlu2 form a functional heteromeric complex, where activation of mGlu2 attenuates signaling through 5-HT_2A_R [23]. *In vivo* electrophysiological studies have demonstrated that the 5-HT_2A/2C_ receptor agonist, (±)-1-(2,5-dimethoxy-4-iodophenyl)-2-aminopropane (DOI), enhances glutamatergic synaptic transmission in the prefrontal cortex of rats [24]. Administration of DOI results in an increase in the expression of c-Fos in several regions of the cortex, including the prefrontal cortex [25]. LY379268 has previously been shown to reverse DOI-induced c-Fos upregulation in the dorsal medial prefrontal cortex (dmPFC, [26]). Here, we tested the effect of LY354740 on DOI-induced increase in c-Fos expression in the rat prelimbic (PrL) and infralimbic (IL) cortex (see Figure 1).

## 2. Results

### 2.1. The Effects of LY354740 and LY379268 Pretreatment on Restraint-Stress-Induced c-Fos Expression

**Figure 2:**
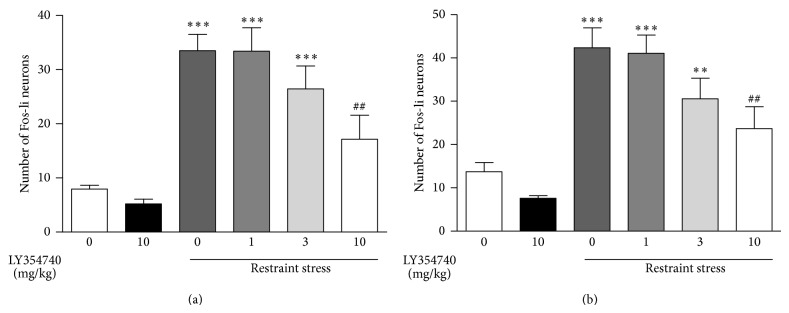
Restraint stress upregulated c-Fos in the PrL (a) and IL (b) cortex. Pretreatment with the mGlu2/3 agonist LY354740 (10 mg/kg, i.p.) attenuated the restraint-induced increase in c-Fos expression in both the PrL and IL cortex. ∗ indicates significantly different from vehicle + home cage; ∗∗∗*P* < 0.001. ^#^ indicates significantly different from vehicle + restraint stress; ^##^ *P* < 0.01. One-way ANOVA with Newman-Keuls post hoc test. Each bar represents the mean (±SEM), *n* = 7-8.

**Figure 3:**
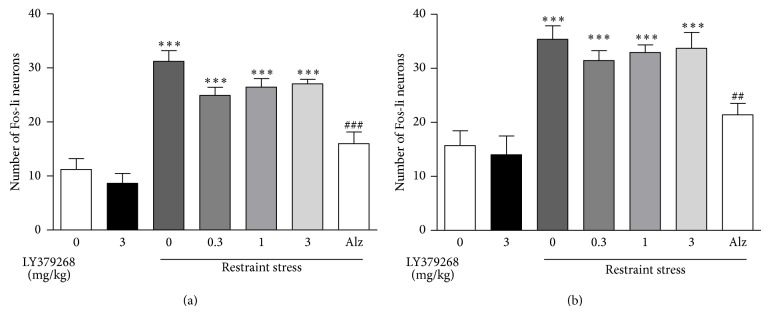
Restraint stress upregulated c-Fos in the PrL (a) and IL (b) cortex. Pretreatment with the mGlu2/3 agonist LY379268 (0.3–3 mg/kg, i.p.) had no effect on the restraint-induced increase in c-Fos expression in the PrL and IL cortex. Pretreatment with alprazolam (3 mg/kg, i.p.) attenuated the c-Fos response in the PrL and IL cortex. ∗ indicates significantly different from vehicle + home cage; ∗∗∗*P* < 0.001. ^#^ indicates significantly different from vehicle + restraint stress; ^##^ *P* < 0.01, ^###^ *P* < 0.001. One-way ANOVA with Newman-Keuls post hoc test. Each bar represents the mean (±SEM), *n* = 7-8.

**Figure 4:**
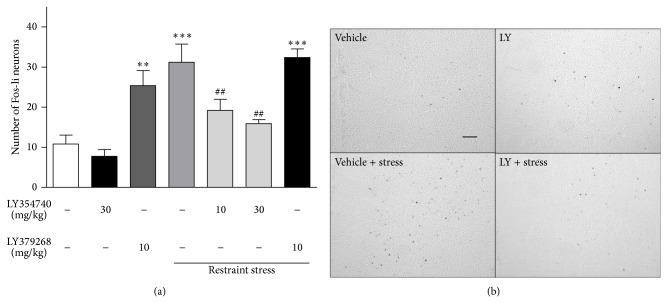
(a) Restraint stress upregulated c-Fos in the PrL cortex. Pretreatment with the mGlu2/3 agonist LY354740 (10 and 30 mg/kg, IP) but not LY379268 (10 mg/kg, i.p.) reversed restraint-induced increase in c-Fos expression in the PrL cortex. ∗∗ indicates significantly different from vehicle + home cage; *P* < 0.01, ∗∗∗*P* < 0.001; ^#^ indicates significantly different from vehicle + restraint stress; ^##^ *P* < 0.01. One-way ANOVA with Newman-Keuls post hoc test. Each bar represents the mean (±SEM), *n* = 7-8. (b) Representative photomicrographs of the prelimbic cortex showing c-Fos-labeled neurons of rats treated either with vehicle (Veh), LY (LY354740 30 mg/kg), vehicle + stress (vehicle + restraint stress), or LY + stress (LY354740 30 mg/kg + restraint stress). Restraint stress increased the number of Fos-positive-labeled cells and pretreatment with LY354740 (30 mg/kg) reversed the effect. LY354740 alone did not enhance Fos immunoreactivity. Scale bar = 100 *μ*m.

The effect of LY354740 and LY379268 was examined in two different but comparable experiments. Restraint stress significantly increased the number of positive cells containing c-Fos immunoreactivity relative to vehicle in both the PrL and IL cortex (*P* < 0.001; Figures 2, 3, and 4). The magnitude of increase was similar between the two areas and between the two experiments (Figures 2 and 3). Prior administration of LY354740 (at 10 and 30, but not 3 mg/kg) significantly reduced the effect of restraint-stress-induced c-Fos protein expression (*P* < 0.01 in both PrL and IL cortex, Figures 2 and 3). Administration of LY354740 alone (10 and 30 mg/kg) had no significant effect on c-Fos expression in any area examined. A representative photomicrograph (Figure 4(b)) from the PrL cortical area shows the effects of stress and the reversal with LY354740 (30 mg/kg) in comparison with the vehicle and LY354740 alone (no stress).

Pretreatment with the LY379268 (0.3, 1, 3, and 10 mg/kg) had no effect on restraint-stress-induced c-Fos protein expression in any of the two areas (Figures 3 and 4(a)). Alprazolam (3 mg/kg, i.p.) significantly reduced the effect of restraint-stress-induced c-Fos protein expression in both PrL and IL (*P* < 0.01) (Figure 3.) While the lower doses of LY379268 alone had no effect on basal c-Fos levels, a higher dose (10 mg/kg) produced a significant upregulation of c-Fos expression to a similar extent as restraint stress (*P* < 0.01), Figure 4(a).

### 2.2. LY354740 Pretreatment Attenuates DOI-Induced c-Fos Expression

**Figure 5:**
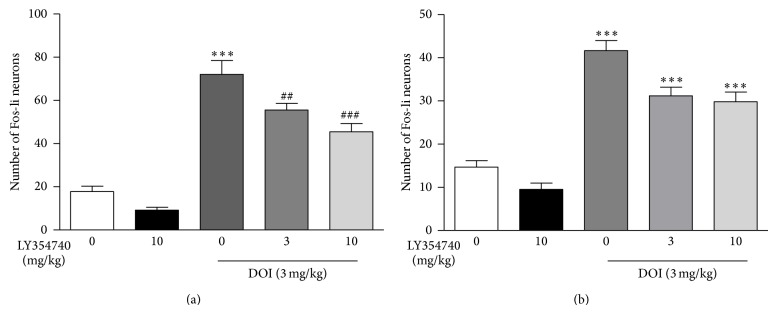
DOI produced an increase in c-Fos in the PrL (a) and IL (b) cortex. Pretreatment with LY354740 (3 and 10 mg/kg) attenuated the DOI-induced increase in the PrL and IL cortex. ∗ indicates significantly different from vehicle; ∗∗∗*P* < 0.001, ^#^ indicates significantly different from DOI; ^##^ *P* < 0.01, ^###^ *P* < 0.001. One-way ANOVA with Newman-Keuls post hoc test. Each bar represents the mean (±SEM), *n* = 7-8.

DOI (3 mg/kg, i.p.) produced a significant increase in c-Fos protein expression in the PrL and IL cortex relative to vehicle-treated animals (*P* < 0.001; Figure 5). The magnitude of increase was much higher than that seen for restraint stress. LY354740 pretreatment at 3 mg/kg, i.p. (*P* < 0.01 in both PrL and IL cortex) and 10 mg/kg, i.p. (*P* < 0.001 in both PrL and IL cortex) significantly reduced the effect of DOI-induced c-Fos protein expression (Figure 5).

## 3. Discussion

In the present study, we examined the effect of the mGluR2/3 agonists LY354740 and LY379268 on immediate early gene (IEG) expression induced by restraint stress. c-Fos is considered a marker of neuronal activity and may have a common mechanism of induction by acute restraint stress and DOI/PCP treatment [28, 29]. Both anxiolytics and antipsychotics have been shown to attenuate IEG expression in cortex induced by stress and the NMDA receptor antagonist phencyclidine (PCP), which are used to model anxiety and schizophrenia, respectively [30–32].

We used restraint stress to induce an anxiety-like state, as this type of stressor has been shown to mediate fear potentiation in the elevated plus maze [33] and robustly increase circulating levels of ACTH and corticosterone [34]. Consistent with previous reports, we observed an increase in c-Fos [18, 19, 21, 35] expression in the forebrain after restraint stress.

It was revealed that while LY354740 had a strong and dose-dependent inhibitory effect on stress-induced c-Fos gene expression, LY379268 did not. These results are in line with behavioral experiments. In the fear-potentiated startle response paradigms, LY354740 demonstrated efficacy in rodents [11] to decrease stress-induced hyperthermia [36] and has even been reported to possess anxiolytic activity in one clinical trial [13]. The ability of LY354740 to attenuate stress-induced c-Fos expression is similar to that seen for the anxiolytic drug class benzodiazepines [22], and Morrow and coworkers demonstrated that the full (Lorazepam) and partial (Bretazenil) benzodiazepine agonists both blocked stress-induced c-Fos levels in the mPFC [37]. Although the focus of the current study was to evaluate effects on c-Fos in the prefrontal cortex, others have shown that LY354740 also can reverse stress-induced c-Fos changes in other areas, including the hippocampal [38]. It is striking that LY379268 had no effects in the same model as LY379268 did not attenuate restraint-stress-induced c-Fos expression in a wide range of doses. By contrast, LY379268 alone caused c-Fos upregulation in PFC to a comparable degree as restraint stress at a higher dose. These results clearly show that LY354740 and LY379268—although highly structurally related—have different pharmacological properties.

The reason for the difference in the anxiolytic-like efficacy between the two compounds is still speculative. However, LY354740 and LY379268 differ somewhat in their *in vitro* potencies at mGlu2 versus mGlu3 receptors [39]. While LY354740 is approximately equipotent at mGlu2 and mGlu3, LY379268 is overall more potent at both receptor subtypes, showing a 5x higher potency at mGlu2 and a 16x higher potency at mGlu3 compared to LY354740 [39]. This difference in *in vitro* profile may account for differences *in vivo*, including brain 2-deoxyglucose utilization studies in the rat, where the LY354740 shows a more general suppression of glucose use across different brain areas [40]. In this study, the authors also noted differences in the overt behavioral responses of the two mGlu2/3 agonists. Interestingly, a recent study showed elevated rat brain 2-DG levels with LY379268 [41]. These results may contribute to the understanding of why LY354740 and LY379268 have different effects in behavioral models of anxiety, such as fear-induced startle [11, 16] and elevated plus maze [11, 12, 14]. In addition, LY379268 has an anxiogenic-like profile at a high dose in the light dark box and open field tests in the rat [17].

Finally, we investigated the interaction between 5-HT_2A_R and mGlu2/3 receptors because recent studies suggest that the effects of mGlu2/3 agonists are partly mediated through 5-HT_2A_ receptors [23, 42]. Previously, LY379268 has been shown to decrease DOI-induced c-Fos expression in dmPFC [26]. We demonstrated the ability of LY354740 to attenuate the DOI-induced c-Fos expression in the rat PrL and IL cortex. Because increased excitation of the PFC has been implicated in the pathophysiology of schizophrenia, the ability of LY354740 and LY379268 to reduce the hallucinogenic drug action in this region could be directly related to its antipsychotic-like efficacy [43]. Antidepressants with anxiolytic properties act in part by blocking the activation of the serotonin 5-HT_2A_ receptors [44], and mice lacking functional 5-HT_2A_ receptors have reduced anxiety-like behaviors [45]. In addition, both compounds show dose-dependent reversal of DOI-induced head twitch behavior in the rodent [24, 46]. We have found similar results with these compounds (Eli Lilly and Company).

Further, the ability of both LY354740 and LY379268 to attenuate DOI-induced c-Fos suggests that this c-Fos response is caused by a different mechanism than that for restraint stress.

In perspective, recent evidence suggests that hyperactivity of the glutamatergic systems in the limbic cortex may contribute to the symptoms of schizophrenia and anxiety [47, 48]. The selective modulation of restraint-stress- and DOI-induced c-Fos expression in the limbic cortex provides additional evidence that mGlu2/3 agonists may serve as an effective therapeutic strategy for preferentially targeting the glutamatergic dysfunction in schizophrenia and anxiety. Thus, the results from our studies provide further insight into the utility of mGlu2/3 orthosteric agonists in the treatment of a variety of psychiatric conditions including schizophrenia and anxiety.

## 4. Experimental Procedures

### 4.1. Animals

All experiments were conducted in accordance with the National Institutes of Health Guide for Care and Use of Laboratory Animals and were approved by the Eli Lilly Institutional Animal Care and Use Committee. Male Sprague Dawley rats, from Harlan, Indianapolis, IN, were housed in groups of four per cage under standard conditions, given free food and water, and maintained on a 12 h light/dark cycle (lights on at 06:00, lights off at 18:00). Animals were acclimatized to the vivarium for at least 6 days prior to the initiation of the study. Studies were performed according to the guidelines of the Animal Care and Use Committee of Eli Lilly & Company.

### 4.2. Drug Treatment

For the DOI studies, rats were prehandled 3-4 days prior to experimentation to minimize stress. On the day of the study, animals were pretreated with vehicle or LY354740 (i.p.) and returned to their home cages. Thirty minutes later, animals were treated with DOI (3 mg/kg, i.p.) or vehicle for DOI. Animals (*n* = 6 to 8 per group) were euthanized by decapitation 2 hours after vehicle or DOI. Whole brains were rapidly removed and immediately immersed in isopentane over dry ice and then stored at −80°C until sectioned.

For the restraint stress studies, rats were prehandled 4-5 days prior to experimentation to minimize stress. On the day of the study, animals were pretreated with vehicle, LY354740 (1, 3, 10, and 30 mg/kg, i.p.), LY379268 (0.3, 1, 3, and 10 mg/kg, i.p.), or alprazolam (3 mg/kg, i.p.) and returned to their home cages. After 30 minutes, animals were restrained in an acrylic flat-bottomed restrainer for 20 minutes. All animals subjected to the restraint stress were housed individually in a quiet room after the removal from the restrainer. Animals (*n* = 7-8 per group) were euthanized by decapitation 2 hours after the onset of restraint. Whole brains were rapidly removed and immediately immersed in isopentane over dry ice and then stored at −80°C until sectioned.

### 4.3. Fos Immunohistochemistry

Coronal sections through the rat prelimbic (PrL, bregma +2.70 mm) and infralimbic (IL, bregma +2.70 mm) brain regions were cut at 14 *μ*m in a cryostat and thaw-mounted onto Superfrost Plus slides (see Figure 1). Sections were allowed to air-dry at room temperature until completely dry and were stored at −20°C until processed. c-Fos single label immunohistochemistry was performed on 14 *μ*m fresh-frozen brain sections.

Slides were brought to room temperature. Slides were immersed in a freshly prepared solution of 4% paraformaldehyde in phosphate-buffered saline for 10 minutes. After being fixed and washed, slides were placed in methanol containing 0.3% hydrogen peroxide for 15 minutes to quench endogenous peroxidases followed by several washes in Tris-buffered saline solution containing 0.05% Tween 20 (TBS-T). Endogenous proteins were blocked by 5-minute incubation in a solution Innogenex Power Block reagent. Excess reagent was carefully suctioned off, and sections were then incubated in a goat anti-Fos IgG (1:750; SC52G, Santa Cruz Biotechnology) in antibody diluent for 90 minutes followed by several washes in TBS-T. Sections were then incubated in a biotinylated horse anti-goat IgG (1:200; Vector Laboratories) in antibody diluent for 30 minutes, followed by several washes in TBS-T. Sections were reacted with avidin-biotin peroxidase complex (Vectastain Elite Kit; Vector Laboratories) for 30 minutes and washed in TBS-T. Fos immunoreactive nuclei were visualized using the Vector VIP substrate kit for peroxidase followed by several washes in water. The slides were then dehydrated and cover-slipped.

### 4.4. Quantification and Data Analysis

A numbered key identified all slides, and quantification was carried out blinded to experimental treatment group. For c-Fos experiments quantification was done using a Sony XC-77 monochrome video camera mounted on a Leica DMR fluorescence microscope. Images were counted live using the image analysis software MCID Elite 6.0. Fos-positive nuclei within 129,000 *μ*m^2^ in the PrL and 100,000 *μ*m^2^ in the IL were counted relative to a threshold based on staining density, target size, and target shape (Figure 1). Counts were made on the left and right sides of 2 sections per animal.

The data was analyzed using a one-way ANOVA followed by Newman-Keuls post hoc test (GraphPad Prism 4.03). The level of significance was set at ∗*P* < 0.05 (compared to vehicle for LY354740 + vehicle for DOI), ^#^ *P* < 0.05 (compared to vehicle for LY354740 + DOI).
